# Recent Advances of Cellulose-Based Hydrogels Combined with Natural Colorants in Smart Food Packaging

**DOI:** 10.3390/gels10120755

**Published:** 2024-11-21

**Authors:** Lan Yang, Qian-Yu Yuan, Ching-Wen Lou, Jia-Horng Lin, Ting-Ting Li

**Affiliations:** 1 School of Textile Science and Engineering, Tiangong University, Tianjin 300387, China; lanyang202210@163.com (L.Y.); yuan_qianyu@163.com (Q.-Y.Y.); cwlou@asia.edu.tw (C.-W.L.); 2 Fujian Key Laboratory of Novel Functional Textile Fibers and Materials, Minjiang University, Fuzhou 350108, China; 3 Department of Bioinformatics and Medical Engineering, Asia University, Taichung City 413305, Taiwan; 4 Advanced Medical Care and Protection Technology Research Center, College of Textile and Clothing, Qingdao University, Qingdao 266071, China; 5 Department of Medical Research, China Medical University Hospital, China Medical University, Taichung City 404333, Taiwan; 6 College of Material and Chemical Engineering, Minjiang University, Fuzhou 350108, China; 7 Advanced Medical Care and Protection Technology Research Center, Department of Fiber and Composite Materials, Feng Chia University, Taichung City 407102, Taiwan; 8 School of Chinese Medicine, China Medical University, Taichung City 404333, Taiwan; 9 Tianjin and Ministry of Education Key Laboratory for Advanced Textile Composite Materials, Tiangong University, Tianjin 300387, China

**Keywords:** cellulose-based hydrogel, natural colors, smart food packaging, food monitoring, pH indicators

## Abstract

Due to the frequent occurrence of food safety problems in recent years, healthy diets are gradually receiving worldwide attention. Chemical pigments are used in smart food packaging because of their bright colors and high visibility. However, due to shortcomings such as carcinogenicity, people are gradually looking for natural pigments to be applied in the field of smart food packaging. In traditional smart food packaging, the indicator and the packaging bag substrate have different degrees of toxicity. Smart food packaging that combines natural colorants and cellulose-based hydrogels is becoming more and more popular with consumers for being natural, non-toxic, environmentally friendly, and renewable. This paper reviews the synthesis methods and characteristics of cellulose-based hydrogels, as well as the common types and characteristics of natural pigments, and discusses the application of natural colorants and cellulose-based hydrogels in food packaging, demonstrating their great potential in smart food packaging.

## 1. Introduction

In recent years, there has been a growing awareness of the importance of food safety issues [1]. Packaging plays a crucial role in both food manufacturing and transportation by protecting food from external environmental factors and extending its shelf life [2,3]. One of the current challenges is to develop methods that can systematically and simply sense and measure the characteristics of the food inside the package. To meet this demand, smart food packaging technology has emerged, using sensors to monitor attributes such as shelf life, quality, safety, pH level, and gas/water vapor permeability, while conveying information about packaged food to consumers [4] or inhibiting the production of bacteria [5,6]. This type of packaging reacts with microbial metabolites and indicators; changes in pH result in color shifts that indicate freshness.

Because cellulose is the most abundant renewable resource on Earth and a major component of plant cell walls, it has been increasingly studied, with recent research focusing on exploring various applications for cellulose-based hydrogels(CBHs), including medical treatment, food packaging [7], and more. Cellulose and its derivatives have the advantages of low cost, high plasticity, excellent water absorption, expansion, biocompatibility, and degradability, making them very suitable for the production of hydrogels [8]. Moreover, cellulose derivatives exhibit strong mechanical strength along with excellent thermal stability; they can endure high temperatures without degrading, while also providing UV protection through their ability to absorb or scatter UV radiation. The carcinogenic risks associated with artificial food coloring agents have raised concerns about their environmental impact as well. While synthetic pigments offer vibrant colors and strong pigmentation capabilities, they may produce toxic compounds during processing [9,10,11] that could lead to health issues like toxicity or cancer [12].

As a result, there is an increasing demand for natural alternatives to replace these artificial dyes. Many natural pigments [13,14,15], such as anthocyanins, carotenoids [16], and curcumin [17], are derived from animal tissues, plant sources, or microorganisms for their effective pH-sensitive discoloration properties, safety profiles, rich availability, and antibacterial effects [18]. Combining these natural colorants with CBHs to create smart packaging films that change color under specific conditions will significantly enhance solutions related to food safety.

For example, anthocyanins of black carrot (ABC) have been added as dyes to a cellulose-chitosan matrix to prepare colorimetric pH indicators for monitoring the deterioration of pasteurized milk. Black carrot anthocyanin (total anthocyanin content is 10 mg/100 mL) was impregnated with chitosan solution to characterize cellulose paper prepared by the sol-gel method. Adding ABC to the chitosan-cellulose membrane increased swelling and water solubility. In food trials, fresh pasteurized milk, after being stored at 20 °C for 48 h, showed a perceptible color change from blue to purple rose, indicating that the coloring film was able to monitor milk freshness [19]. J. Chumee et al. [13] prepared a natural composite of agar, sodium alginate, and polyvinyl alcohol mixed with cabbage anthocyanin extract as a natural polymer to prepare colorimetric biofilm sensors for smart packaging of fresh pork. QIN et al. [20] found that adding anthocyanin-rich wolfberry extract and betacyanidin-rich dragon fruit extract to starch/PVA film was more suitable for monitoring pork freshness. In other studies, a chitosan (CS)/methylcellulose (MC) matrix was combined with the anthocyanins of the mature fruits of phyllophilus (PR) to prepare a smart film with biological activity and pH responsiveness. The smart film was pink in acidic pH solutions and light yellow in alkaline pH solutions. It showed strong antioxidant and antibacterial activity against *Staphylococcus aureus*, *Pseudomonas aeruginosa*, *Escherichia coli* and other common food-borne pathogens. The smart membrane also showed potential for monitoring fish freshness.

In this review, we classified cellulose-based hydrogels and natural pigments, introduced the structure, categories, and functions of cellulose-based hydrogels and natural pigments in detail, and reviewed the application of smart materials combining natural pigments and cellulose-based hydrogels in smart packaging for different food types. This paper provides ideas for further promoting the development of materials combining natural pigments and cellulose-based hydrogels in intelligent packaging.

## 2. Cellulose-Based Hydrogel

### 2.1. Cellulose-Based Hydrogel Synthesis

**Figure 1:**
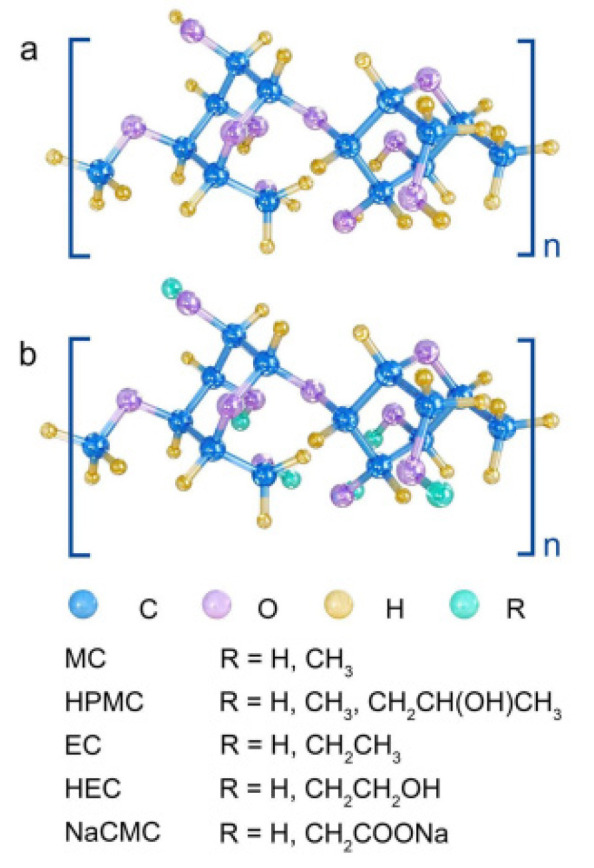
(**a**) The repeating unit of cellulose, also known as “cellulobiose”. (**b**) Repeating units of cellulose derivatives. Substituent “R” for methyl cellulose (MC), hydroxypropyl methyl cellulose (HPMC), ethyl cellulose (EC), hydroxyethyl cellulose (HEC), and sodium carboxymethyl cellulose (NaCMC).

Cellulose serves as the primary constituent of natural fibers found in various sources like plants, cotton, and hemp. It is classified as a polysaccharide composed of glucose units linked by 1,4-beta-glucosidic bonds. This compound is characterized by its lack of odor and taste, along with being insoluble in water and most organic solvents. Certain bacteria, including Acetobacter xylintans, are capable of producing cellulose as well [21]. Although microbial or bacterial cellulose (BC) shares the same chemical composition as plant cellulose (PC), they differ in their macromolecular structures and physical characteristics [22]. Cellulose is difficult to dissolve due to its hydrophilicity and strong intermolecular and intramolecular hydrogen bonds, as well as van der Waals forces [23]. Chemical modification of cellulose usually involves esterification or etherification of its hydroxyl group, resulting in cellulose derivatives such as methyl cellulose (MC), hydroxypropyl methyl cellulose (HPMC), ethyl cellulose (EC), hydroxyethyl cellulose (HEC), and sodium carboxymethyl cellulose (NaCMC). The structure of these derivatives is shown in Figure 1. These cellulose derivatives are considered environmentally friendly as they can be broken down by various bacteria and fungi found in air, water, and soil that produce cellulase-specific enzymes (known as cellulases) [24]. The environmental friendliness of cellulose and its derivatives stems from their ability to be decomposed by several microorganisms capable of synthesizing these specific enzymes. The process of cellulose biodegradation has been thoroughly investigated, leading to a decrease in molecular weight, diminished mechanical strength, and enhanced solubility. Furthermore, the reduction in crystallinity along with increased water solubility can contribute to faster rates of cellulose biodegradation [25]. Being the most prevalent natural polymer on our planet, cellulose is not only biodegradable but also renewable and recyclable, making it widely applicable in areas such as hydrogels and aerogels.

Hydrogel consists of a three-dimensional network formed by hydrophilic polymers capable of holding significant amounts of water and nutrients over extended periods. It can absorb, expand, and release substantial quantities of water and biological fluids while exhibiting excellent pressure resistance, biocompatibility, and environmental safety. The presence of hydrogen bonds and van der Waals forces gives cellulose or its derivatives a hydrophilic surface. This allows for crosslinking into a three-dimensional network of CBHs with various metals, organic compounds, and polymers through mechanisms such as hydrogen bonding, covalent bonding, or ionic interactions. The material can hold significant amounts of water or aqueous solutions, including physiological solutions, without dissolving or compromising its structural integrity. It is used in various sectors such as the food industry, agriculture, water purification, and biomedicine. Zhao et al. [26] utilized chitosan derived from chitin to create hydrogels based on chitosan and employed glucan as a crosslinking agent for drug delivery systems. The resulting hydrogel features a porous structure with excellent expansion capabilities, supports cell proliferation effectively, and aids in wound healing. Another study implemented a two-step method to fabricate carboxymethyl CBHs through molding followed by acidification, yielding a material that is suitable for self-healing applications and can also function as a sealant or for gastric mucosa repair [27].

There are three ways to synthesize CBHs: physical crosslinking, chemical crosslinking, and interpenetrating polymerization.

Due to the abundance of hydroxyl groups in cellulose’s molecular structure, hydrogels derived from cellulose can be created through physical crosslinking via hydrogen bonding, resulting in a network formation. When employing this method for hydrogel preparation, the molecular chains of cellulose establish network structures through various interactions, such as hydrogen bonds, ionic forces, molecular entanglement, or hydrophobic interactions, leading to the creation of insoluble physical hydrogels. The interactions among these physical cross-linked cellulose gel molecules are reversible; thus, alterations in physical conditions can disrupt the network structure. Recently, this method has attracted more and more attention because it shows much richer properties and application potential than polymers prepared by chemical crosslinking. In addition, physical crosslinking does not require the use of any chemical reagents, so it is considered a simple and cost-effective way to prepare hydrogels. This crosslinking process can be facilitated by a variety of techniques, including heating or cooling polymer solutions, complex condensation processes, freeze-thaw cycles, hydrogen bonding, ionic interactions, and self-assembly strategies. The primary techniques for achieving physical crosslinking include solution-based methods and freeze-thaw approaches. The mechanism behind gelation involves hydrophobic binding between macromolecules that contain methoxyl groups. At lower temperatures, polymer chains within the solution become intertwined with one another while interacting with water molecules. As the temperature rises, macromolecules gradually lose moisture until associations between polymers occur due to their hydrophobic nature, resulting in a hydrogel network formation. The sol-gel transition temperature is influenced by both the degree of substitution present in cellulose ether and the salt concentration added during processing [28]. For instance, utilizing solvent casting technology allows methyl cellulose and chitosan to dissolve within an appropriate solvent. Subsequent storage of this mixture in a dryer containing anhydrous calcium chloride facilitates drying; it is then combined with anthocyanins extracted from mature blue fruit leaves, ultimately producing an active smart film responsive to pH changes. This film demonstrated significant antioxidant properties and antibacterial efficacy against common food-borne pathogens like *Staphylococcus aureus*, *Pseudomonas aeruginosa*, *Escherichia coli*, among others [20].

While physical crosslinked hydrogels are straightforward to produce and do not necessitate the use of crosslinking agents or chemical modifiers, they exhibit certain limitations in terms of strength. Additionally, physical crosslinking is reversible; under specific conditions such as mechanical stress, these hydrogels may flow and can degrade unpredictably [29]. In contrast to the fluid nature of physically formed hydrogels, chemically crosslinked hydrogels establish stable and rigid networks by linking cellulose chains together. The process of chemical crosslinking involves creating a network structure among macromolecular chains through the formation of chemical bonds facilitated by light, heat, or other media along with various crosslinking agents. Typically, in chemically synthesized hydrogels, attention is given to the interactions between polymers and their respective crosslinkers. The properties of these hydrogels—particularly their mechanical strength—are influenced by the specific functional groups present in the crosslinking agent. Under extreme conditions like low pH levels and high temperatures—with methanol added as a quencher—polymers containing hydroxyl groups can be linked using glutaraldehyde. Furthermore, polysaccharides can undergo crosslinking with reagents such as 1,6-hexamethylenediisocyanate or dialkyl sulfone to create hydrogel structures. These reagents serve to ensure that particular polymers or functional groups form interconnected networks within the gels [30]. CBHs produced via chemical methods tend to have more stable molecular configurations and enhanced swelling capabilities. Common techniques for this include radical homopolymerization or copolymerization triggered by chemicals and radiation methods.

Polymerization serves as an additional crosslinking method for the creation of hydrogels, which includes techniques such as bulk polymerization, solution polymerization, and irradiation polymerization. The primary process involves only a monomer and an initiator, with the rate and extent of polymerization being dependent on the concentration of the monomer. In solution copolymerization involving two polymers, it is essential to arrange them in a hydrophilic manner—whether random, structured, blocky, or alternating—to form network configurations [31]. Moreover, blocks formed through copolymerization can create hydrogels when applied in situ; these hydrogels are both biodegradable and biocompatible. The rapid gelation time, combined with interactions between synthetic hydrophilic polymers and biopolymers during irradiation polymerization, leads to the formation of macromolecular monomers. CBHs produced via this technology exhibit a dual-component network structure that provides high swelling ratios alongside favorable mechanical properties. Polymerization methods may also be advantageous in more complex processes due to their efficiency with regard to time and product output [32].

Furthermore, whether through chemical or physical means, crosslinking remains crucial for maintaining the integrity of hydrogel’s three-dimensional polymer networks during fabrication. Polymerization technologies hold promising applications within biomedical tissue engineering as well as adsorption and separation fields.

### 2.2. Characteristics of Cellulose-Based Hydrogels

Due to its three-dimensional network structure and hydrophilic polymer chain, cellulose hydrogels can absorb and retain a large amount of water in the gap structure, and have good water absorption, which makes cellulose hydrogels widely used in many fields, including medical field, food industry, agriculture, water purification, and so on. The water absorption of cellulose-based hydrogels is also related to the preparation method. For example, carboxymethyl cellulose and cellulose hydrogels prepared in a sodium hydroxide/urea/water system exhibit superabsorbency and self-healing behavior by chemical crosslinking methods. CBHs has shown clear advantages in some specific areas, such as wound dressings and drug delivery systems. One of their remarkable characteristics is their expansibility, which directly affects their water absorption ability and moisturizing effect. CBHs can absorb large amounts of water, up to 1000 times its dry weight, making it ideal for use in food packaging, especially when it comes to humidity control [33,34]. For example, in the study of swelling properties, carboxymethyl cellulose (CMC)-based hydrogel film prepared with polyvinyl alcohol (PVA) as a binder showed excellent hygroscopic and water-absorbing ability and was regarded as a promising fruit and vegetable packaging material.

Additionally, CBHs demonstrate remarkable mechanical characteristics along with significant strength. In food packaging contexts, cellulose-based hydrogel materials serve as innovative intelligent packaging solutions where durability against external forces during transport and storage is vital. For instance, in scenarios where moisture levels vary among different food types, CBHs must retain their structural integrity while functioning effectively across various temperature and humidity conditions. A highly elastic hydrogel film composed of CMC (carboxymethyl cellulose), PVA, polyethylenimine (PEI), and tannic acid (TA) has shown impressive tensile strain capabilities up to 400% without failure for food packaging uses [35].

Studies have shown that there are many factors affecting the mechanical strength of CBH, including the polymerization degree of cellulose chain, the type and concentration of crosslinking agent, the selected solvent, the use of filler, and the preparation process [36]. Therefore, due to their excellent mechanical properties, these materials show a wide range of application prospects in the field of materials science and engineering and play an important role.

### 2.3. Cellulose-Based Hydrogel Application Classification

**Figure 2:**
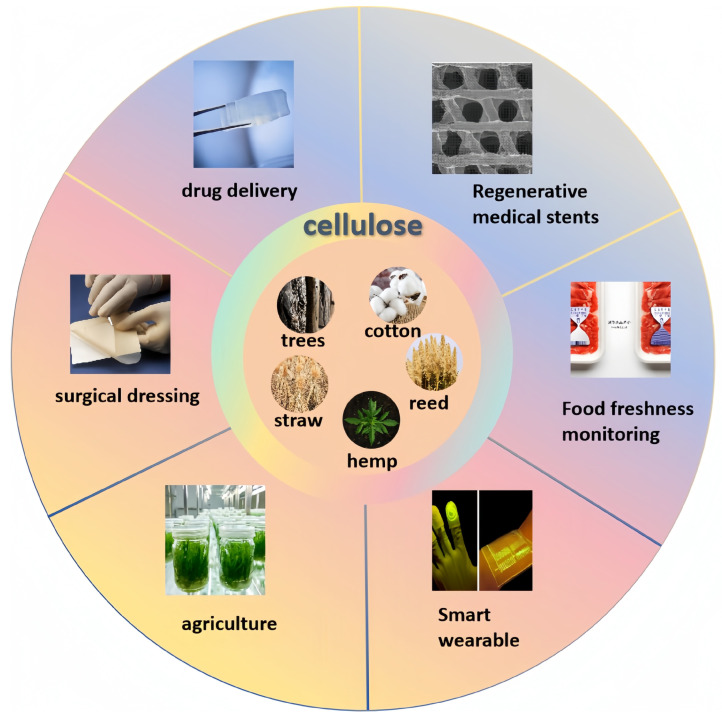
Sources of cellulose and applications of CHBs.

Cellulose serves as a precursor for cellulose hydrogels and is characterized by its low cost, abundant availability, excellent biocompatibility, and certain reactivity to external stimuli. Consequently, CBHs have diverse applications that extend from traditional uses as water-absorbing materials to more advanced fields such as biomedical applications and smart packaging solutions [8]. Figure 2 shows the source of cellulose and the applications of cellulose-based hydrogels.

#### 2.3.1. Wound Dressing

Hydrogel medical dressings feature a three-dimensional network structure that can retain up to 96% water content, creating a moist environment on the wound’s surface [37]. Unlike traditional sterile gauze, these dressings do not adhere to the wound, which helps alleviate pain and minimizes fluid loss while protecting the skin from infection. They also allow for breathability, preventing scab and scar formation that could lead to secondary injuries, and their humid conditions promote faster healing [37]. This makes them an ideal choice for next-generation wound care materials.

To date, various hydrogel dressings have been patented and commercialized using either synthetic or natural polymers or combinations thereof. Notable recent patents include those detailing in situ gel formation (for example, based on sprayable formulations [38] and incorporated nanoparticles [39]) and exploring radiation crosslinking as a method of stabilization that enables the creation of sterile crosslinked hydrogel films in one step [40,41]. The most advanced versions often incorporate antimicrobial agents like silver ions.

It is essential to recognize that existing products are typically designed for specific types of wounds and may require additional secondary dressings. Therefore, there are many studies involving the development of novel wound dressings with better performance, such as the preparation of highly efficient wound healing methods using carboxymethyl cellulose/quaternized starch in the presence of antibacterial natural cinnamon essential oil as nanoemulsion [42]. Hydrogels are prime candidates for this purpose—whether in sheet form or amorphous structures—and their transparency offers an added benefit in such applications.

#### 2.3.2. Drug Release

Research on drug release is advancing towards achieving high efficiency, rapid action, prolonged effects, minimal toxicity and side effects, and reduced dosage requirements, as well as ease of production, transportation, storage, carrying, and administration. Cellulose hydrogels have become an innovative continuous release carrier due to their porous three-dimensional structure formed by adjustable crosslinking density and pore size. Their open pores and large specific surface area make it possible to effectively load and release drug molecules when external environmental conditions are changed. Such hydrogels are receiving increasing attention in drug release control.

When utilizing hydrogels for regulating drug delivery, the loading process can occur either after crosslinking or concurrently during network formation [43]. Additionally, bioactive molecules may be covalently or physically attached to the polymer network to further modulate the rate of release. Smart hydrogels are particularly advantageous for managing both temporal and spatial aspects of a drug’s release profile, as changes in mesh size within the hydrogel network—triggered by physiological variables such as pH levels, temperature fluctuations, and ionic strength—can influence this process [44]. The controlled release mechanism of the oral drug delivery system depends on the significant pH changes encountered when transitioning from the gastric environment to the intestinal environment; cellulose-based polyelectrolyte hydrogels (such as those containing sodium carboxymethyl cellulose) prove especially suitable for these applications. 

#### 2.3.3. Regenerative Medical Stents

Tissue-engineered cellulose hydrogels feature a sufficiently large pore structure and three-dimensional space. Their high water content makes them biocompatible, while their mechanical properties are similar to soft tissue. These hydrogels typically allow the incorporation of cells and bioactive molecules during the gel process. While cells may have difficulty adhering to highly hydrophilic surfaces [45,46], the volume or surface chemistry of these hydrogels can be easily modified using extracellular matrix (ECM) components, thereby enhancing cell adhesion and specific cell functions [47]. Mimicking the flexibility of natural tissues, they serve as organ-like structures that provide living cells with a supportive environment for survival and nutrient storage.

Consequently, hydrogels present an ideal platform for creating biomimetic scaffolds aimed at tissue regeneration. A recent study suggests pretreating cellulose-based scaffolds with cellulase prior to implantation to control the degradation behavior of cellulose in vivo [48]. The researchers also noted that glucose, a byproduct of cellulose degradation, provides nutrients to cells, illustrating the benefits of utilizing cellulose over other synthetic or natural polymers in tissue engineering applications. In addition, the bulk chemistry of cellulose-based hydrogels is easier to modify than cellulose sponges and fabrics.

#### 2.3.4. Agriculture

Cellulose-based hydrogels have attracted great attention in agriculture because of their biodegradability, renewability, non-toxicity, and ecological friendliness [49]. These cellulose-based hydrogels are used in various aspects of agricultural systems, including improving soil permeability, density, structure, water evaporation rate, filtration efficiency, and texture. They also facilitate the transport and release of agrochemicals while promoting plant growth and stimulating germination in arid conditions. This ensures a consistent supply of agrochemicals to crops while minimizing environmental pollution from these substances [50].

For instance, hydrogels with high water retention capabilities can reduce overall water usage and optimize resource management in agricultural practices. As they expand, these materials transition from a glassy state to a rubbery state that allows them to retain water, even under considerable compression. If there is a humidity difference between the interior and exterior of the material, expanded hydrogels can gradually release stored water through diffusion-driven mechanisms. Additionally, when further molecules are incorporated into the polymer network during synthesis alongside water addition, they can be released in a controlled manner over time.

To address issues related to water scarcity in arid or desert regions globally, dry gels (i.e., dehydrated hydrogels) could be mixed as powders or pellets into soil near plant roots. These dry gels may also contain nutrients or botanical extracts. When irrigation occurs, the hydrogels absorb moisture, which is then gradually released along with nutrients back into the soil as needed, maintaining prolonged soil moisture levels. This method significantly conserves water by reducing rapid loss through evaporation and drainage while reallocating cultivated resources for other uses.

#### 2.3.5. Smart Material

Cellulose-based hydrogels are increasingly used in the manufacture of smart devices due to their excellent biocompatibility, high adsorption capacity of cells and small molecules, and low interfacial tension at the gel–water interface. This class of smart materials exhibits unique properties, including excellent mechanical strength and biocompatibility. Composite hydrogels that combine cellulose with carbon nanotubes have a wide range of applications in conductive materials, wearable electronics, structural health monitoring systems, smart textiles, liquid leak detection devices, and strain sensors [51], as well as thermistors and humidity or steam sensors. In addition, the hydrogel film can also act as a highly sensitive nanosensor, reflecting information through pH changes.

## 3. Overview of the Natural Colorants Can Combine with CBHs

CBHs have shown significant promise as pH sensors and smart packaging solutions due to their ability to respond to variations in environmental pH and monitor food freshness, among other applications. By integrating natural pigments—both of which are renewable materials—these hydrogels enhance water absorption while minimizing food waste. Additionally, incorporating smart color-changing features into CBHs for packaging can substantially reduce food waste. Natural pigments are usually derived from the roots and stems of natural plants, flowers, fruits and animals, microorganisms, etc., with high safety and low toxicity characteristics, usually have a variety of nutritional and pharmacological effects, and have great practical application value in the market. Natural pigments are divided into three categories according to their source: plant pigments, such as chlorophyll in green leaves (green), carotene in carrots (orange) [52], lycopene in tomatoes (red), etc.; animal pigments, such as heme in muscle (red), astaxanthin in shrimp shell (red), etc.; microbial pigments, such as soybean curd surface monascus pigment (red), and so on. Pigments can also be classified according to structure, divided into porphyrin derivatives, isoprene derivatives, polyphenol derivatives, ketone derivatives, quinone derivatives, and six other categories.

### 3.1. Anthocyanin

Anthocyanins, the most abundant secondary plant metabolites in the plant family, are water-soluble natural colors that are extensively present in plants in nature. In nature, more than 635 anthocyanins have been found [53,54]. Anthocyanins are important components in various substances including six the following types: pelargonidin, cyanidin, delphinidin, peonidin, morning glory, and hibiscus. In their natural state, anthocyanins exist in the form of glycosides, meaning they exist as anthocyanin glucosides and rarely as free anthocyanins [55]. Due to their powerful antioxidant properties, anthocyanins also help protect plants from UV damage [56]. In vitro studies have shown that they inhibit the replication of syncytial virus, parainfluenza virus, cytomegalovirus simplex, adenovirus, and rotavirus [57]. Anthocyanins are mainly used in the area of food coloring, but can also be used in dyes, pharmaceuticals, and cosmetics.

Anthocyanins are glycosylated derivatives of 2-pHenylbenzopyran, which have polyhydroxy and polymethoxy groups. They usually have molecular weights ranging from 400 to 1200 and contain two benzyl rings. Anthocyanins occur mainly in plant vesicles that are converted from chlorophyll. These compounds typically have a single unit of glycoside, but most also have two or more sugars attached in multiple positions or in the form of oligosaccharide side chains. Free anthocyanins are rare in occurrence. The higher the anthocyanin content, the darker the food’s color, and the color shifts with pH changes. At pH 7, it is red, between pH 7–8, it is purple, and above pH 11, it turns blue.

There are two main reasons for promoting research on anthocyanins. Firstly, their role in preserving the sensory properties of food is crucial to the production of food technology. Secondly, anthocyanins promote health via numerous pathways and confer several benefits. Anthocyanins are one of the primary pigments found in fruits, stems, flowers, roots, and leaves, and they usually dissolve evenly in vacuolar solutions of epidermal cells. However, the concentration of anthocyanins can vary due to the influence of various environmental factors, including agronomic and genetic factors, light intensity and type, and temperature. Additionally, fruits with the same genetic background can possess different quantities and compositions of anthocyanins [58].

### 3.2. Betalains

Betaine is a water-soluble nitrogenous pigment and a derivative of pyridine. It consists of two forms, betalain and betaxanthin. It is generally found in the flowers and fruits of plants and some asexual reproductive organs. Because of its prominent and uniform color at pH 3–7, beet is widely used as a food colorant [9]. In addition, beetroot possesses antioxidant and antibacterial properties. It can undergo structural changes and turn yellow in an alkaline environment, exhibiting stronger dyeing ability, higher dyeing intensity, and water solubility [59]. Compared to anthocyanins, betalains are suitable as a pigment for neutral or low-acidic foods and have been extensively studied for their pH indicators in food packaging [60].

Red and yellow betalains are ammonium couplings of betulinic acid rain cyclic dihydroxyphenylalanine (cDOPA) glucoside and amino acids or amines, respectively [61]. They can be further classified as red/purple (maximum absorption about 540 nm) [62] and yellow/orange (maximum absorption about 480 nm). Depending on the substitution position (hydroxyl (-OH) group at the 5 or 6 position of betalains) and the mode of substitution (glycosylation, acylation, and esterification), betalains can be classified into different types such as betalains (the most abundant constituents in plant sources), isocyanin, chloropHyll, root green, gastrin, and amaranth, with different structures and colors [63]. As a natural pigment, betalains are somewhat color-independent of pH and are more stable than anthocyanins [64].

### 3.3. Curcumin

Curcumin has been widely used as a spice in food since ancient times and has the advantages of being highly beneficial and abundant, as well as having a variety of biological properties, such as antiviral, antioxidant, antitumor, antimicrobial, and choleretic properties [65]. It is one of the largest selling natural colorants in the world. Curcumin is widely used as a bioactive ingredient with free radical scavenging, anticancer, antiangiogenic, antidiabetic, and antimicrobial properties in food additives, herbal and functional foods, flavors, and colors [66,67].

Curcumin molecules feature numerous double bonds and active functional groups, including phenolic hydroxyl and carbonyl, which showcase substantial physiological activity. Curcumin represents a distinctive diketone pigment found in the plant realm, comprising two adjacent methylated phenols and a β-diketone. The β-diketone structure may display an enol-keto tautomeric conformation, with spectroscopic evidence verifying curcumin’s primary presence in the enol form, both in a solid state and a solution.

Curcumin as a natural yellow acidic phenolic pigment has a wide range of applications and great potential for development. As a representative of natural pigments, curcumin has a stronger safety than synthetic pigments and is currently one of the world’s top seven largest-selling natural food colors; it is mainly used for coloring candy, wrapping powder, and convenient rice noodle food. Because of its anti-cancer, antioxidant, and other physiological activities, it can be used as medicine to treat diseases. Curcumin capsules have entered the health care market, with clinical studies showing that they have a certain therapeutic effect against cardiovascular system diseases, gastrointestinal diseases, and cancer. Curcumin can be used in cosmetics and health care products; however, because curcumin is difficult to dissolve in water, its bioavailability is extremely low, which restricts the scope of its pharmacological effects [67].

### 3.4. Carotenoids

Carotenoids are the most studied natural pigments and belong to the fat-soluble group, being found in most fruits and vegetables, plants, algae [68], and photosynthetic bacteria [69,70]. More than 650 different types exist in nature [71,72]. They are available in orange, yellow, and red colors as their chromophore consists mainly of a coupled double interchain [70]. They are the main source of vitamin A in the body [72] and also have antioxidant, immunomodulatory, anticancer, and aging-delaying properties.

Carotenoids are isoprenoid compounds that contain a basic structural skeleton of 40 carbon atoms. They generally consist of two molecules of twenty carbon atoms connected by a bis-coumaroyl bisphosphate tail, from which different types of carotenoids are derived. These carotenoids all contain many conjugated double bonds [73]. They can be divided into two main groups according to whether they contain oxygen atoms in the molecular structure: oxygen-free carotenoids (e.g., lycopene, α-carotene, β-carotene, etc.) [74] and oxygen-containing carotenoids (e.g., lutein [75], anther xanthophylls, astaxanthin [76], etc.), which include both double-epoxide and monoepoxide. According to the derived structure at the end of the carbon skeleton, they can be categorized into open-ring carotenoids (e.g., lycopene, octahydrolycopene) and ring carotenoids (e.g., α-carotene, β-carotene, etc.).

Since animals cannot synthesize carotenoids, their presence is a result of either direct accumulation of carotenoids in food or partial modification by metabolic reactions [77]. Carotenoid patterns in animals therefore provide the key to tracing food chains and metabolic pathways [78]. For example, pink salmon flesh and the color of many birds’ feathers are due to carotenoids, whereas most microorganisms and algae resynthesize carotenoids from isoprene diphosphate via phytoene and lycopene, which are then altered to form other derivatives (deoxyxylulose pathway) [72].

## 4. Application of CBHs with Natural Colorants in Smart Food Packaging

CBHs can be synthesized through a variety of pathways, including cellulose and its derivatives such as methylcellulose(MC), hydroxypropyl cellulose(HPC), hydroxypropyl methylcellulose (HPMC), and CMC. Due to their improved chemical structure, these derivatives improve the solubility, viscosity, and other properties of cellulose, making them highly valuable in many applications [79,80]. In addition, certain ester derivatives, such as triacetate, phthalate, methyl hydroxypropyl phthalate, and hydroxypropyl phthalate methacrylate, can also be used in the production of CBHs [81,82]. Each derivative has its own unique characteristics, and the properties of CBH can be customized according to specific needs.

CBHs can be achieved by binding to cellulose and other polymers, or by forming polyelectrolyte complexes. For example, CMC can be combined with PVA, PEI, and TA to form a hydrogel film with high elasticity and adaptability. In addition, smart colorimeters that combine natural dyes with CBHs are increasingly used in smart food packaging solutions such as meat, seafood, fruits, vegetables, and various dairy products. This technology helps consumers assess food quality promptly while ensuring safety [7]. This section will explore different applications involving the combination of natural colorants with CBHs in intelligent food packaging.

### 4.1. Meat

Meat and meat products are rich in natural nutrients, are a great source of amino acids, vitamins, minerals, and high-quality protein, and play a key role in the human diet [83]. However, these beneficial products fall into the category of perishable foods because they are rich in nutrients and contain unsaturated fats as well as high water activity. Therefore, meat is very sensitive to microbial growth and oxidative spoilage [84]. Therefore, the use of smart packaging films can show the freshness of meat products, extend shelf life, and improve food quality and safety during transportation and storage [85,86].

Anthocyanins are often used to make meat biosensing films to assess the freshness of food. In one study, Chayavanich et al. mixed cornstarch and gelatin with anthocyanins extracted from carrots to create a pH-sensitive membrane. In the pH range of 2 to 12, the membrane color rapidly changes from orange to gray-purple. The sensor film demonstrated good stability and high sensitivity over 14 days. Therefore, it is recommended to use this pH indicator membrane to monitor the freshness of chicken meat [87].

In another study on CBHs, J. Chumee et al. [13] made a colorimetric biofilm sensor using AGAR, sodium alginate, and polyvinyl alcohol as natural complex polymers to extract anthocyanins from red cabbage. When the sensor is mixed with anthocyanin extract, it can be used to detect the degree of pork spoilage. When total volatile basic nitrogen (TVB-N) exceeds a set threshold, the color changes from pink to orange. In addition, Chi et al. [88] developed a smart film based on κ-carrageenan/hydroxypropyl methylcellulose and anthocyanins extracted from grape skin powder. The team found that when the pH value reached 7.0, the film appeared blue-green, at which point the TVB-N value had exceeded 14.63 mg/100 g. This preparation can be implemented for monitoring pork freshness. Similarly, Choi et al. [89] developed colorimetric pH indicator membranes employing agar, potato starch, and 27 naturally derived dyes. The pH values of the indicator membranes, which contained anthocyanins, exhibited considerable absorption peaks in the UV-visible region. It demonstrated a color change from red to green when the pork became unsuitable for consumption. Therefore, it suggests that these indicator membranes can accurately assess pork freshness. Sun et al. [90] produced pH-sensitive membranes using hydroxypropyl methylcellulose/k-carrageenan and anthocyanins extracted from kiwifruit pomace. At 8% anthocyanin content, the film demonstrated considerable sensitivity to gaseous amines, leading to a change in color. When the level of TVB-N surpassed the limit and reached 25.69 mg/100 g following 40 h of storage, the film that was enriched with anthocyanin displayed a bluish-black hue, signaling that the pork had gone bad. Therefore, the perceptible color alteration of films deployed on pork can be suitably employed for tracking freshness, microbial growth, and other chemical transformations in other foods that are abundant in animal protein. Esfahani et al. [91] prepared a smart edible film using tapioca starch and pomegranate peel powder and employed this film for lamb packaging, where the color of the film changed from red to green at 25 °C. The color change of the smart film allowed the detection of the total volatile basic nitrogen (TVBN) content in the meat, making it possible to use this smart active film as an indicator of lamb freshness. In addition to this, in another study, Alizadeh-Sani et al. [92] prepared a biopolymer matrix containing methylcellulose and chitosan nanofibers and combined them with saffron anthocyanins. It was shown that the sown films containing saffron anthocyanins could track the freshness of lamb meat and responded well to different pH values. It can reflect the quality and freshness information of meat in real time. Thus, the visible color changes of the film applied to meat can be appropriately used to monitor the freshness of other animal protein-rich foods, microbial growth, and other chemical changes. Table 1 shows the application of CBHs with natural colorants in meat intelligent packaging.

### 4.2. Sea Food

Since fish and other seafood products are extremely fragile and highly perishable products, concerns about their safety and quality have prompted the adoption of several traceability methods to assess the freshness of seafood products [93]. Deterioration of seafood products is usually caused by a variety of chemical reactions or microbial actions [94,95]. Therefore, by monitoring dimethylamine, trimethylamine, and ammonia levels, it is possible to determine the freshness of seafood products in the early stages of spoilage. The increase in volatile nitrogen levels can be regarded as a potential indicator of fish spoilage; the freshness of seafood products can be assessed by pH sensing membranes, which monitor changes in pH caused by alkaline volatile gases produced by the fish and visibly change color.

You et al. [96] incorporated blackcurrant anthocyanins into a konjac glucomannan (KGM)/carboxymethyl cellulose (CMC) composite film to improve the structure and properties of the film. The results showed that anthocyanins were uniformly dispersed in the matrix and could change the mechanical properties of the films. Used in smart food packaging, the film—which changed from pink to light yellow at pH 2–12—can be used for freshness monitoring of tilapia. In addition to this, Yan et al. [95] prepared pH indicator films by mixing the natural polymer chitosan (CH) with natural dyes from butterfly pudding extract (BP). The films were determined by testing various properties of the films, including their microstructure and mechanical properties. Changes in total volatile basic nitrogen (TVB-N) content during the deterioration of tilapia affected the pH change, which visually affected the film color. The color of the film ranged from pinkish purple to yellow at pH 1–14, and the film showed a clear color change from violet-blue to dark green during the fish preservation process. The results indicate that the film is a smart packaging film that can be used to monitor the freshness of fish. Tavakoli et al. [97] developed potential pH-responsive films by incorporating a mixture of anthocyanins and phycocyanin into a composite gelatin/soy polysaccharide matrix. The results showed that the smart film changed from dark orange to dark blue with the degree of fish spoilage; it was considered a promising sensor for nondestructive monitoring of fish freshness. In another experiment, visual pH sensing membranes based on starch, polyvinyl alcohol, and glycerol containing curcumin (CR) and anthocyanin (ATH) were prepared to monitor the freshness of fish in real time and non-destructively, with a range of pH color changes from 5 to 11. Through the results of the color stability test, the authors showed that the composite membranes incorporating curcumin were the most stable and had the best correlation with the freshness of the fish, and therefore were able to monitor the spoilage of fish in real time [98]. In order to study the application of natural pigment smart pH membranes in fish freshness monitoring, GE and other researchers [99] used oxidized chitin nanocrystals (O-ChNCs) and gelatin as substrates, combined with black rice bran anthocyanins (BACNs), and successfully prepared a green nanocomposite membrane with pH sensitivity and antioxidant properties. Although the addition of black rice bran anthocyanins resulted in a decrease in the mechanical properties of the film, it significantly improved its anti-ultraviolet and anti-oxidation capabilities. When prawns and scallops were monitored for spoilage, the film changed color from purple to grayish blue or brown. Because the film has strong antioxidant activity, it can delay the deterioration process of packaging products to a certain extent. Dong et al. [100] developed a colorimetric sensing film with high mechanical strength and hydrophobicity based on cellulose and naphthoquinone dyes to monitor shrimp freshness in real time. The performance of the colorimetric sensing membrane was evaluated with shrimp at 20, 4, and −20 °C. The sensing membrane clearly changed from rose red to purple and then to blue-violet in the pH range of 5–12, which provided good indications of spoilage and holds great promise for visual monitoring of shrimp freshness. Chen et al. [101] successfully immobilized red cabbage anthocyanins in chitosan (CS)/oxidized chitin nanocrystal (cyanate) composites by hydrogen bonding reaction to form a cohesive membrane structure. The freshness of scallop and shrimp was monitored in real time, and the smart film successfully distinguished three stages of product freshness (fresh, medium-fresh, and spoiled) by a significant color change (red-pink-blue-green) in response to ammonia vapors and acidic/alkaline environments within a short period of time. Ma et al. [102] prepared smart films by adding curcumin (Cur) to a mixed matrix of Tara gum and polyvinyl alcohol (PVA) to study the surface; the addition of curcumin resulted in a relaxation of the film structure and a decrease in the light transmission, but an increase in the surface wettability. Spoilage experiments were also conducted on shrimp to evaluate the practical application of the film. Shrimp produce volatile alkaline nitrogen during spoilage, including ammonia, dimethylamine, trimethylamine, etc.; therefore, the film increased with the increase in alkaline nitrogen, and the pH increased and changed from yellow to orange-red. Table 2 shows the application of CBHs with natural colorants in seafood intelligent packaging.

### 4.3. Others

Food industry items, including milk, fruits, vegetables, pastries, and other food products, are rich in polysaccharides or proteins. To protect the food from physical, chemical, or biological stresses in the environment and thereby improve its quality and prolong its shelf-life, the use of intelligent packaging instead of traditional food packaging has become a key research goal in recent years. These new packaging systems are an effective way to extend or maintain shelf life. Therefore, the use of natural coloring to monitor food freshness is one of the safer, healthier, and more environmentally friendly solutions available in smart packaging.

Milk is a nutrient-dense food, composed of high-quality proteins, sugars, fats, vitamins, and enzymes, and has a slightly acidic pH range of 6.4 to 6.8. Although a complete food, milk is also highly prone to contamination and thus requires proper preservation and storage techniques. The pasteurization process is often employed to preserve milk, accompanied by the application of several chemical preservatives, including bronopol, potassium dichromate, hydrogen peroxide, and azidiol. Although these processes eliminate bacterial contamination, there are still some issues posed by the health risks associated with these preservation methods [103]. Therefore, many scholars have investigated different smart packages to determine whether milk has spoiled in a simple and clear manner. Roy et al. [103] produced functional smart films incorporating gelatin and carrageenan, utilizing shikonin and propolis. The color-responsive film and shikonin solution exhibited a remarkable pH-responsive color change over a broad pH range of 2–12. Fresh milk has a pH level of 6.6, and a decrease to 5.5 signifies that it has started to spoil, while a pH of 4.5 implies that it has completely deteriorated. The membrane devised by Roy et al. bolsters food safety and extends the longevity of food provisions through quality monitoring. Liu et al. [104] designed a κ-carrageenan combined with Lycium barbarum extract as a wide pH-sensitive colorimetric membrane for milk freshness monitoring. The colorimetric film changed from grey to dark pink, which showed the deterioration behavior of milk, and was reversible, indicating its great potential for freshness monitoring.

The preservation of cheese is an increasing problem due to microbial contamination and infestation, so Bandyopadhyay et al. prepared a polyvinylpyrrolidone-carboxymethylcellulose-bacterial cellulose-guar gum-based membrane with red kale anthocyanin as a pH indicator membrane that can detect the freshness of cheese [105]. Junior et al. [106] prepared smart time-temperature indicator membranes based on gibberellic acid polysaccharide/polyvinyl alcohol blended with red kale anthocyanin to indirectly indicate changes in food quality. The interaction between the indicating membrane and the milk caused a change in color. When the milk deteriorated, the color of the indicator film changed from dark gray to dark pink, clearly indicating that the milk was no longer fresh. This phenomenon reflects the change in pH due to spoilage, which changes the chemical properties of food, and can be applied to smart packaging to monitor the freshness of milk. Tirtashi et al. [107] developed a colorimetric pH indicator to monitor spoilage of pasteurized milk by incorporating anthocyanins extracted from black carrots into a chitosan/cellulose film. The indicator film showed a distinct color change from pink to khaki in the pH range of 2–11. Stability experiments showed that the film remained acceptably stable after storage at 20 °C for one month.

An important indicator of fruit freshness is ripeness, which is often a difficult indicator for consumers to estimate. Spoilage of fruit results from a reduction in the degree of inedible quality. While indicators of food spoilage may be more relevant to fish and meat, fruit also exhibits certain indicators of spoilage due to different external factors. Spoilage of fruit is caused by external factors including temperature, humidity, gases, and the surrounding atmosphere. Food spoilage can be detected using chemical sensors, including pH sensing membranes, for noninvasive and real-time monitoring of fruit freshness [108]. In order to monitor fresh blueberries, Li et al. [109] prepared a pH-sensitive film containing blueberry anthocyanin extract, which was based on a pectin-sodium alginate-xanthan gum composite. The effects of different concentrations of blueberry anthocyanin extracts on the microstructure and physical properties of the films were comprehensively evaluated. The results showed that the film was significantly responsive to changes in pH value, showing different and easily recognizable colors under different pH conditions. As the blueberries deteriorated, the color of the film gradually changed from purple to light pink. To ensure food quality, El-Shall et al. [110] used methylcellulose (CMC), pomegranate anthocyanin extract (PAE), and SCOBY (BCCC) bacterial cellulose at 1–15% concentration to develop a film that is antioxidant, antibacterial, UV-resistant, pH-sensitive, edible, and flexible. As a potent antioxidant, PAE is effective in inhibiting a wide range of pathogenic Gram-negative and -positive bacteria, whether in solution form or embedded in composite membranes, with inhibition diameters between 20 and 30 mm. By using this smart packaging technology, it is possible to extend the shelf life of red grapes and plums by 25 days and maintain better quality compared to leaving the fruit in its unpackaged state. Zong et al. [111] developed smart food packaging by embedding purple sweet potato polyphenol extract (SPS) in a starch/gelatin film with pH between 3 and 10. Using this membrane, they were able to test the freshness of the fresh wood mushrooms. During storage, the color of the smart film gradually changes from green to purplish gray and eventually yellow, indicating that the material has the potential to be used as a smart packaging solution for other foods in the future. Table 3 shows examples of CBHs and natural colorants used in other smart packaging applications.

## 5. Challenges and Future Perspectives

In recent years, the emergence of demand for healthy, fresh, and safe food has brought opportunities and challenges to the production institutions and industries of smart food packaging. Smart packaging solutions and indicator labels that incorporate natural colorants with CBHs can effectively monitor the freshness and quality of packaged foods. These intelligent packages are capable of detecting changes in food conditions, such as spoilage, ripeness, and microbial activity, displaying visual color shifts to communicate information regarding food quality.

Natural fruits and vegetables offer a variety of pigments suitable for use in the food industry and smart packaging applications as alternatives to synthetic dyes. These healthy pigments align well with safe, non-toxic packaging materials. Thus, combining natural colorants with CBHs addresses consumer health needs while promoting environmentally friendly practices. Beyond being safer for human consumption, these materials also allow for evaluating packaged food quality based on the color changes exhibited by natural pigments, further enhancing food safety.

Numerous studies have indicated that CBHs possess superior mechanical properties, barrier capabilities, and water retention compared to other options. They have proven effective at prolonging the shelf life of various foods like fruits, vegetables, and meats by acting as barriers against moisture, oxygen, and microbial growth. Research has highlighted their suitability across different food packaging environments. Efforts are being made to strengthen the application of natural colorants in this field. It should be noted that, compared to industrial gel materials, the production cost of cellulose hydrogels may be higher than that of traditional plastic materials. Therefore, in order to ensure high profits, many companies choose cheaper materials. In addition, due to the special nature of the source of cellulose-based hydrogels, ensuring consistent production costs and performance remains a challenge, and processing conditions need to be further addressed.

## Figures and Tables

**Table 1:** Application Of CBHs With Natural Colorants in meat intelligent packaging.

Product Category	Based on Film/Other Active Components	Natural Pigment Source	pH Change Range	Color Change Range	Color Change Range
Chicken	Corn starch, gelatin	Carrot anthocyanins	2–12	From orange to gray-purple	[87]
Pork	AGAR, sodium alginate and polyvinyl alcohol	Red cabbage anthocyanins	2–11	From pink to orange	[13]
Pork	κ-carrageenan, hydroxypropyl methyl cellulose	Grape skin powder anthocyanins	2–7	From pink to purple to turquoise	[88]
Pork	AGAR, potato starch	anthocyanins	2–10	From red to green	[89]
Pork	Hydroxypropyl methylcellulose, k-carrageenan	Anthocyanins extracted from kiwifruit pomace	2–12	From red to purple	[90]
Mutton	Tapioca starch	Pomegranate peel powder anthocyanins		From red to gree	[91]
Mutton	Methyl cellulose, chitosan nanofibers	Saffron anthocyanins	1–14	From purple to green/yellow	[92]

**Table 2:** Application of CBHs with natural colorants in seafood intelligent packaging.

Product Category	Based on Film/Other Active Components	Natural Pigment Source	pH Change Range	Color Change Range	Color Change Range
tilapia	Konjac glucomannan (KGM)/Carboxymethyl cellulose (CMC)	Black currant anthocyanins	2–12	From pink to pale yellow	[96]
tilapia	Chitosan, a natural polymer	Butterfly pudding extract	1–14	From purplish blue to dark green	[95]
fish	Compound gelatin, soybean polysaccharide matrix	Anthocyanin and phycocyanin mixture	5–8	From dark orange to dark blue	[97]
fish	Starch, polyvinyl alcohol, glycerin	Curcumin (CR) and anthocyanin (ATH)	5–11	From dark gray to yellow	[98]
Shrimp and hairtail	Oxidized chitin nanocrystals (O-ChNCs), gelatin	Black rice bran anthocyanins (BACNs)	2–12	From purple to grayish blue or brown	[99]
shrimp	cellulose	Naphthoquinone dye	5–12	From rosy red to purple to blue-purple	[100]
shrimp	Chitosan (CS), oxidized chitine nanocrystals (cyanate)	Red cabbage anthocyanins	3–10	From red to pink to blue to green	[101]
shrimp	Tara gum, polyvinyl alcohol (PVA)	Curcumin (Cur)		From yellow to orange-red	[102]

**Table 3:** Applications of CBHs and natural colorants in other smart packaging.

Product Category	Based on Film/Other Active Components	Natural Pigment Source	pH Change Range	Color Change Range	Color Change Range
milk	Gelatin/carrageenan	alkannin	2–12	From red to blue	[103]
milk	κ-Carrageenan	Wolfberry extract	2–10	From grey to dark pink	[104]
cheese	Polyvinylpyrrolidone—Carboxymethyl cellulose—bacterial cellulose—Guar gum	Red cabbage anthocyanins		From red to blue	[105]
milk	Red peach, polyvinyl alcohol	Red cabbage anthocyanins	8–5	From dark gray to dark pink	[106]
milk	Chitosan, cellulose film	Black carrot anthocyanins	2–11	From pink to khaki	[107]
blueberry	Pectin Sodium alginate Xanthan gum composite film (PAX)	Blueberry anthocyanin extract (BAEs)	2–13	From purple to light pink	[109]
Red grapes and plums	Methyl cellulose (CMC), and SCOBY (BCCC) (1–15%) bacterial cellulose	Pomegranate anthocyanins	2–12	From red to green	[110]
Needle mushroom	Starch/gelatin film	Purple sweet potato polyphenol extract	3–10	From green to purplish gray to yellow	[111]

## Data Availability

No new data were created or analyzed in this study. Data sharing is not applicable to this article.

## References

[1] Moustafa H., Hemida M.H., Nour M.A., Abou-Kandil A.I. (2024). Intelligent packaging films based on two-dimensional nanomaterials for food safety and quality monitoring: Future insights and roadblocks. J. Thermoplast. Compos..

[2] Li T.T., Li Y., W L.M. (2019). Research progress of intelligent packaging indicator in food industry. Food Res. Dev..

[3] Yam K.L. (2012). Emerging Food Packaging Technologies.

[4] Gholampour S., Jalali H., Zhiani R., Rashidi H., Motavalizadehkakhky A. (2021). Biogenic amines to tune the LSPR adsorption peak of gold NPs for intelligent packaging application. Inorg. Chem. Commun..

[5] Moustafa H., Hemida M.H., Shemis M.A., Dufresne A., Morsy M. (2023). Functionalized GO nanoplatelets with folic acid as a novel material for boosting humidity sensing of chitosan/PVA nanocomposites for active food packaging. Surf. Interfaces.

[6] Moustafa H., Ahmed E.M., Morsy M. (2022). Bio-based antibacterial packaging from decorated bagasse papers with natural rosin and synthesised GO-Ag nanoparticles. Mater. Technol..

[7] Singh A.K., Itkor P., Lee Y.S. (2023). State-of-the-Art Insights and Potential Applications of Cellulose-Based Hydrogels in Food Packaging: Advances towards Sustainable Trends. Gels.

[8] Hasan A.M.A., Abdel-Raouf E.S. (2019). Cellulose-Based Superabsorbent Hydrogels. HASAN, AMA, and ABDEL-RAOUF, ME-S. Cellulose-Based Superabsorbent Hydrogels.

[9] Rodriguez-Amaya D.B. (2019). Update on natural food pigments—A mini-review on carotenoids, anthocyanins, and betalains. Food Res. Int..

[10] Tsai C.F., Kuo C.H., Shih Y.C. (2015). Determination of 20 synthetic dyes in chili powders and syrup-preserved fruits by liquid chromatography/tandem mass spectrometry. J. Food Drug Anal..

[11] Yadav S., Tiwari K.S., Gupta C., Tiwari M.K., Khan A., Sonkar S.P. (2023). A brief review on natural dyes, pigments: Recent advances and future perspectives. Results Chem..

[12] Zhang H., Zhan J., Su K., Zhang Y. (2006). A kind of potential food additive produced by *Streptomyces coelicolor*: Characteristics of blue pigment and identification of a novel compound, λ-actinorhodin. Food Chem..

[13] Chumee J., Kumpun S., Nimanong N., Banditaubol N., Ohama P. (2022). Colorimetric biofifilm sensor with anthocyanin for moni-toring fresh pork spoilage. Mater. Today Proc..

[14] Zheng Y., Li X., Huang Y., Li H., Chen L., Liu X. (2022). Two colorimetric films based on chitin whiskers and sodium alginate/gelatin incorporated with anthocyanins for monitoring food freshness. Food Hydrocoll..

[15] Garavand F. (2022). Application of Red Cabbage Anthocyanins as pH-Sensitive Pigments in Smart Food Packaging and Sensors. Polymers.

[16] Li Z.H., Li Y., Wang X., Wu K.X., Wang Y.W. (2021). Application of intelligent indicator in food packaging. J. Wuhan Univ. Light Ind..

[17] Zheng H., Jiang H.T., Wang L.X., Liu Y., Wang W.T., Li D., Li L. (2020). Preparation and application of anthocyanin/curcumin smart labels. Packag. Eng..

[18] Rotariu L., Lagarde F., Jaffrezic-Renault N., Bala C. (2016). Electrochemical biosensors for fast detection of food contaminants—Trends and perspective. TrAC Trends Anal. Chem..

[19] Hu X., Shou B., Yang L., Li L., Ren H.T., Lin J.H., Lou C.W., Li T.T. (2023). Antimicrobial photodynamic therapy encapsulation technology: Frontier exploration and application prospects of novel antimicrobial technology. Chem. Eng. J..

[20] Qin Y., Xu F., Yuan L., Hu H., Yao X., Liu J. (2020). Comparison of the physical and functional properties of starch/polyvinyl alcohol films containing anthocyanins and/or betacyanins—ScienceDirect. Int. J. Biol. Macromol..

[21] Ross P., Mayer R., Benziman M. (1991). Cellulose biosynthesis and function in bacteria. Microbiol. Rev..

[22] Czaja W.K., Young D.J., Kawecki M., Brown R.M. (2007). The future prospects of microbial cellulose in biomedical applications. Biomacromolecules.

[23] Luo X., Zhang L. (2010). New solvents and functional materials prepared from cellulose solutions in alkali/urea aqueous system. Food Res. Int..

[24] Tomšič B., Simončič B., Orel B., Vilčnik A., Spreizer H. (2007). Biodegradability of cellulose fabric modified by imidazolidinone. Carbohydr. Polym..

[25] Miyamoto T., Takahashi S., Ito H., Inagaki H., Noishiki Y. (1989). Tissue biocompatibility of cellulose and its derivatives. J. Biomed. Mater. Res..

[26] Zhao Y., Zhang X., Wang Y., Wu Z., An J., Lu Z. (2014). In situ cross-linked polysaccharide hydrogel as extracellular matrix mimics for antibiotics delivery. Carbohydr. Polym..

[27] Zheng W.J., Gao J., Wei Z., Zhou J., Chen Y.M. (2015). Facile fabrication of self-healing carboxymethyl cellulose hydrogels. Eur. Polym. J..

[28] Sannino A., Demitri C., Madaghiele M. (2009). Biodegradable Cellulose-based Hydrogels: Design and Applications. Materials.

[29] Te Nijenhuis K. (2007). On the nature of crosslinks in thermoreversible gels. Polym. Bull..

[30] Hennick W.E., Nostrum C.F. (2012). Novel crosslinking methods to design hydrogels. Adv. Drug Deliv. Rev..

[31] Mohite P.B., Adhav S.S. (2017). A hydrogels: Methods of preparation and applications. Int. J. Adv. Pharm..

[32] Zainal S.H., Mohd N.H., Suhaili N., Anuar F.H., Lazim A.M., Othaman R. (2021). Preparation of cellulose-based hydrogel: A review. J. Mater. Res. Technol..

[33] Ren H.T., Cao W.B., Qin J., Cai C.C., Li D.S., Li T.T., Lou C.W., Lin J.H. (2024). Enhanced removal of tetracycline by sandwich layer composite membrane based on the synergistic effect of photocatalysis and adsorption. Sep. Purif. Technol..

[34] Klein M., Poverenov E. (2020). Natural biopolymer-based hydrogels for use in food and agriculture. J. Sci. Food Agric..

[35] Zhao Y., Zhou S., Xia X., Tan M., Lv Y., Cheng Y., Tao Y., Lu J., Du J., Wang H. (2022). High-performance carboxymethyl cellulose-based hydrogel film for food packaging and preservation system. Int. J. Biol. Macromol..

[36] Oprea M., Voicu S.I. (2020). Recent advances in composites based on cellulose derivatives for biomedical applications. Carbohydr. Polym..

[37] Winter G.D. (1962). Formation of the scab and the rate of epithelization of superficial wounds in the skin of the young domestic pig. Nature.

[38] Sinko P.J., Stein S., Menjoge A.R., Gunaseelan S., Anumolu S.N., Navath R. (2011). Dressing Compositions and Methods. Patent.

[39] St. John J.V., Moro D.G. (2008). Hydrogel Wound Dressing and Biomaterials Formed in Situ and Their Uses. Patent.

[40] Yang Z., Rao Z., Yue L., Yang S., Zhu N. (2008). Medical Hydrogel Wound Dressing and Preparation Method Thereof. Patent.

[41] Burd A., Tsang M.W. (2008). Wound Healing Dressings and Methods of Manufacturing the Same. Patent.

[42] Moustafa H., Nasr H.E., Youssef A.M. (2024). Development of antibacterial carboxymethyl cellulose/quaternized starch bionanocomposites based on cinnamon essential oil nanoemulsion for wound healing applications. Biomass Convers. Bior..

[43] Drury J.L., Mooney D.J. (2003). Hydrogels for tissue engineering: Scaffold design variables and applications. Biomaterials.

[44] Peppas N.A. (1997). Hydrogels and drug delivery. Curr. Opin. Colloid. Interface Sci..

[45] Hu X., Zhang H., Wang Y., Shiu B.C., Lin J.H., Zhang S., Lou C.W., Li T.T. (2022). Synergistic antibacterial strategy based on photodynamic therapy: Progress and perspectives. Chem. Eng. J..

[46] Nguyen K.T., West J.L. (2002). Photopolymerizable hydrogels for tissue engineering applications. Biomaterials.

[47] Seliktar D. (2005). Extracellular Stimulation in Tissue Engineering. Ann. N. Y. Acad. Sci..

[48] Entcheva E., Bien H., Yin L., Chung C.Y., Farrell M., Kostov Y. (2004). Functional cardiac cell constructs on cellulose-based scaffolding. Biomaterials.

[49] Varaprasad K., Raghavendra G.M., Jayaramudu T., Yallapu M.M., Sadiku R. (2017). A mini review on hydrogels classification and recent developments in miscellaneous applications. Mater. Sci. Eng. C.

[50] Elbarbary A.M., El-Rehim H.A.A., El-Sawy N.M., Hegazy E.-S.A., Soliman E.-S.A. (2017). Radiation induced crosslinking of polyacrylamide incorporated low molecular weights natural polymers for possible use in the agricultural applications. Carbohydr. Polym..

[51] Sannino A., Pappadà S., Giotta L., Valli L., Maffezzoli A. (2007). Spin coating cellulose derivatives on quartz crystal microbalance plates to obtain hydrogel-based fast sensors and actuators. J. Appl. Polym. Sci..

[52] Perrin F., Brahem M., Dubois-Laurent C., Huet S., Jourdan M., Geoffriau E., Gagné S. (2016). Differential pigment accumulation in carrot leaves and roots during two growing periods. J. Agric. Food Chem..

[53] Zhang Y., Butelli E., Martin C. (2014). Engineering anthocyanin biosynthesis in plants. Curr. Opin. Plant Biol..

[54] Smeriglio A., Barreca D., Bellocco E., Trombetta D. (2016). Chemistry, Pharmacology and Health Benefits of Anthocyanins. Phytother. Res..

[55] Bueno J.M., Sáez-Plaza P., Ramos-Escudero F., Jiménez A.M., Fett R., Asuero A.G. (2012). Analysis and Antioxidant Capacity of Anthocyanin Pigments. Part II: Chemical Structure, Color, and Intake of Anthocyanins. Crit. Rev. Anal. Chem..

[56] Sunil L., Shetty N.P. (2022). Biosynthesis and regulation of anthocyanin pathway genes. Appl. Microbiol. Biotechnol..

[57] Pour P.M., Fakhri S., Asgary S., Farzaei M.H., Echeverria J. (2019). The Signaling Pathways, and Therapeutic Targets of Antiviral Agents: Focusing on the Antiviral Approaches and Clinical Perspectives of Anthocyanins in the Management of Viral Diseases. Front. Pharmacol..

[58] Oladzadabbasabadi N., Nafchi A.M., Ghasemlou M. (2022). Natural anthocyanins: Sources, extraction, characterization, and suitability for smart packaging. Food Packag. Shelf Life.

[59] Calva-Estrada S., Jiménez-Fernández M., Lugo-Cervantes E. (2022). Betalains and their applications in food: The current state of processing, stability and future opportunities in the industry. Food Chem..

[60] Abedi-Firoozjah R., Parandi E., Heydari M., Kolahdouz-Nasiri A., Bahraminejad M., Mohammadi R., Rouhi M., Garavand F. (2023). Betalains as promising natural colorants in smart/active food packaging. Food Chem..

[61] Schliemann V.W. (2003). Recent advances in betalain research. Phytochemistry.

[62] Li Y.C., Liu S.Y., Meng F.B. (2020). Comparative review and the recent progress in detection technologies of meat product adultera-tion. Compr. Rev. Food Sci. Food Saf..

[63] Fu Y., Shi J., Xie S.Y., Zhang T.Y., Aluko R.E. (2021). Red beetroot betalains: Perspectives on extraction, processing, and potential health benefits. J. Agric. Food Chem..

[64] Tanaka Y., Sasaki N., Ohmiya A. (2008). Biosynthesis of plant pigments: Anthocyanins, betalains and carotenoids. Plant J..

[65] Roy S., Priyadarshi R., Ezati P., Rhim J.W. (2022). Curcumin and its uses in active and smart food packaging applications—A compre-hensive review. Food Chem..

[66] Fereydouni N., Movaffagh J., Amiri N., Darroudi S., Darroudi M. (2021). Synthesis of nano-fibers containing nano-curcumin in zein corn protein and its physicochemical and biological characteristics. Sci. Rep..

[67] Moghadamtousi S.Z., Kadir H.A., Hassandarvish P. (2014). A Review on Antibacterial, Antiviral, and Antifungal Activity of Curcumin. Biomed. Res. Int..

[68] Chen Z., Wu W., Wen Y., Zhang L., Wu Y., Farid M.S., El-Seedi H.R., Capanoglu E., Zhao C. (2023). Recent advances of natural pigments from algae. Food Prod. Process. Nutr..

[69] Milani A., Basirnejad M., Shahbazi S., Bolhassani A. (2017). Carotenoids: Biochemistry, pharmacology and treatment. Br. J. Pharmacol..

[70] Eggersdorfer M., Wyss A. (2018). Carotenoids in human nutrition and health. Arch. Biochem. Biophys..

[71] Khoo H.E., Prasad K.N., Kong K.W., Jiang Y., Ismail A. (2011). Carotenoids and their isomers: Color pigments in fruits and vegeta-bles. Molecules.

[72] Matsuno T. (2001). Aquatic animal carotenoids. Fish. Sci..

[73] Ito M., Yamano Y., Tode C., Wada A. (2010). Carotenoid synthesis: Retrospect and recent progress. Arch. Biochem. Biophys..

[74] Fu X., Cheng S., Feng C., Kang M., Huang B., Jiang Y., Duan X., Grierson D., Yang Z. (2019). Lycopene cyclases determine high α-/β-carotene ratio and increased carotenoids in bananas ripening at high temperatures. Food Chem..

[75] Estévez-Santiago R., Beltrán-de-Miguel B., Olmedilla-Alonso B. (2016). Assessment of dietary lutein, zeaxanthin and lycopene intakes and sources in the Spanish survey of dietary intake (2009–2010). Int. J. Food Sci. Nutr..

[76] Maoka T., Kuwahara T., Narita M. (2014). Carotenoids of Sea Angels *Clione limacina* and *Paedoclione doliiformis* from the Perspective of the Food Chain. Mar. Drugs.

[77] Mortensen A. (2006). Carotenoids and other pigments as natural colorants. Pure Appl. Chem..

[78] Matsuno T., Hirao S. (1989). Marine carotenoids. Marine Biogenic Lipids, Fats, and Oils.

[79] Akter M., Bhattacharjee M., Dhar A.K., Rahman F.B.A., Haque S., Rashid T.U., Kabir S.M.F. (2021). Cellulose-Based Hydrogels for Wastewater Treatment: A Concise Review. Gels.

[80] Sun B., Zhang M., Shen J., He Z., Fatehi P., Ni Y. (2019). Applications of Cellulose-based Materials in Sustained Drug Delivery Systems. Curr. Med. Chem..

[81] Kabir S.M.F., Sikdar P.P., Haque B., Bhuiyan M.A.R., Ali A., Islam M.N. (2018). Cellulose-based hydrogel materials: Chemistry, prop-erties and their prospective applications. Prog. Biomater..

[82] Arca H.C., Mosquera-Giraldo L.I., Bi V., Xu D., Taylor L.S., Edgar K.J. (2018). Pharmaceutical Applications of Cellulose Ethers and Cellulose Ether Esters. Biomacromolecules.

[83] Yehia H.M., Al-Masoud A.H., Elkhadragy M.F., Korany S.M., Nada H.M.S., Albaridi N.A., Alzahrani A.A., Al-Dagal M.M. (2021). Improving the Quality and Safety of Fresh Camel Meat Contaminated with *Campylobacter jejuni* Using Citrox, Chitosan, and Vacuum Packaging to Extend Shelf Life. Animals.

[84] Vergara H., Cózar A., Rubio N. (2021). Lamb meat burgers shelf life: Effect of the addition of different forms of rose-mary (*Rosmarinus officinalis* L.). CyTA—J. Food.

[85] Chen S., Brahma S., Mackay J. (2020). The role of smart packaging system in food supply chain. J. Food Sci..

[86] Mahmood K., Kamilah H., Alias A.K., Ariffin F., Nafchi A.M. (2021). Functionalization of electrospun fish gelatin mats with bioactive agents: Comparative effect on morphology, thermo-mechanical, antioxidant, antimicrobial properties, and bread shelf stability. Food Sci. Nutr..

[87] Chayavanich K., Thiraphibundet P., Imyim A. (2019). Biocompatible film sensors containing red radish extract for meat spoilage observation. Spectrochim. Acta A.

[88] Chi W., Cao L., Sun G., Meng F.S., Zhang C.J., Li J., Wang L.J. (2019). Developing a highly pH-sensitive k-carrageenan-based intelligent film incorporating grape skin powder via a cleaner process. J. Clean. Prod..

[89] Choi A.I., Lee B.J.Y., Lacroix C.M., Han D.J. (2017). Intelligent pH indicator film composed of agar/potato starch and anthocyanin extracts from purple sweet potato—ScienceDirect. Food Chem..

[90] Sun G., Chi W., Xu S., Wang L. (2020). Developing a simultaneously antioxidant and pH-responsive κ-carrageenan/hydroxypropyl methylcellulose film blended with *Prunus maackii* extract—ScienceDirect. Int. J. Biol. Macromol..

[91] Esfahani A., Nafchi A.M., Baghaei H. (2022). Fabrication and characterization of a smart film based on cassava starch and pomegranate peel powder for monitoring lamb meat freshness. Food Sci. Nutr..

[92] Alizadeh-Sani M., Tavassoli M., McClements D.J., Hamishehkar H. (2021). Multifunctional halochromic packaging materials: Saffron petal anthocyanin loaded-chitosan nanofiber/methyl cellulose matrices. Food Hydrocoll..

[93] Morsy M.K., Zór K., Kostesha N. (2016). Development and validation of a colorimetric sensor array for fish spoilage monitoring. Food Control.

[94] Wu C., Sun J., Zheng P., Kang X., Chen M., Li Y., Ge Y., Hu Y., Pang J. (2019). Preparation of an intelligent film based on chi-tosan/oxidized chitin nanocrystals incorporating black rice bran anthocyanins for seafood spoilage monitoring. Carbohydr. Polym..

[95] Yan J., Cui R., Qin Y., Li L., Yuan M. (2021). A pH indicator film based on chitosan and butterfly pudding extract for monitoring fish freshness. Int. J. Biol. Macromol. Struct. Funct. Interact..

[96] You P., Wang L., Zhou N., Yang Y., Pang J. (2022). A pH-intelligent response fish packaging film: Konjac glucomannan/carboxymethyl cellulose/blackcurrant anthocyanin antibacterial composite film. Int. J. Biol. Macromol..

[97] Tavakoli S., Mubango E., Tian L. (2023). Novel intelligent films containing anthocyanin and phycocyanin for nondestructively trac-ing fish spoilage. Food Chem..

[98] Chen H.Z., Zhang M., Bhandari B., Yang C.H. (2020). Novel pH-Sensitive Films Containing Curcumin and Anthocyanins to Monitor Fish Freshness. Food Hydrocoll..

[99] Ge Y., Li Y., Bai Y., Yuan C., Wu C., Hu Y. (2019). Intelligent gelatin/oxidized chitin nanocrystals nanocomposite films containing black rice bran anthocyanins for fish freshness monitorings. Int. J. Biol. Macromol..

[100] Dong H., Ling Z., Zhang X., Zhang X., Xu F. (2020). Smart colorimetric sensing films with high mechanical strength and hydrophobic properties for visual monitoring of shrimp and pork freshness. Sens. Actuators B Chem..

[101] Chen M., Yan T., Huang J., Zhou Y., Hu Y. (2021). Fabrication of halochromic smart films by immobilizing red cabbage anthocyanins into chi-tosan/oxidized-chitin nanocrystals composites for real-time hairtail and shrimp freshness monitoring. Int. J. Biol. Macromol. Struct. Funct. Interact..

[102] Ma Q., Du L., Wang L. (2017). Tara gum/polyvinyl alcohol-based colorimetric NH3 indicator films incorporating curcumin for intel-ligent packaging. Sens. Actuators B Chem..

[103] Roy S., Rhim J.W. (2021). Preparation of Gelatin/Carrageenan-Based Color-Indicator Film Integrated with Shikonin and Propolis for Smart Food Packaging Applications. ACS Appl. Bio Mater..

[104] Liu J., Wang H., Guo M., Li L., Chen M., Jiang S., Li X., Jiang S. (2019). Extract from *Lycium ruthenicum* Murr. Incorporating κ-carrageenan colorimetric film with a wide pH–sensing range for food freshness monitoring. Food Hydrocoll..

[105] Bandyopadhyay S., Saha N., Zandraa O. (2020). Essential Oil Based PVP-CMC-BC-GG Functional Hydrogel Sachet for ‘Cheese’: Its Shelf Life Confirmed with Anthocyanin (Isolated from Red Cabbage) Bio Stickers. Foods.

[106] Pereira V.A., Arruda I.N.Q., Stefani R. (2015). Active chitosan/PVA films with anthocyanins from *Brassica oleraceae* (Red Cabbage) as Time–Temperature Indicators for application in intelligent food packaging. Food Hydrocoll..

[107] Tirtashi F.E., Moradi M., Tajik H., Forough M., Ezati P., Kuswandi B. (2019). Cellulose/chitosan pH-responsive indicator incorporated with carrot anthocyanins for intelligent food packaging. Int. J. Biol. Macromol..

[108] Alam A.U., Rathi P., Beshai H., Sarabha G.K., Deen M.J. (2021). Fruit Quality Monitoring with Smart Packaging. Sensors.

[109] Li Y., Hu Z., Huo R. (2023). Preparation of an indicator film based on pectin, sodium alginate, and xanthan gum containing blue-berry anthocyanin extract and its application in blueberry freshness monitoring. Heliyon.

[110] El-Shall F.N., Al-Shemy M.T., Dawwam G.E. (2023). Multifunction smart nanocomposite film for food packaging based on carbox-ymethyl cellulose/Kombucha SCOBY/pomegranate anthocyanin pigment. Int. J. Biol. Macromol..

[111] Zong Z., Chen H., Wu W., Fang X., Niu B., Gao H., Liu M., Farag M.A. (2023). Preparation and characterization of a novel intelligent starch/gelatin binary film containing purple sweet potato anthocyanins for *Flammulina velutipes* mushroom freshness monitoring. Food Chem..

